# CTLA-4 +49A/G Polymorphism Increases the Susceptibility to Bladder Cancer in Chinese Han Participants: A Case-Control Study

**DOI:** 10.1155/2020/8143158

**Published:** 2020-12-01

**Authors:** Fei Mao, Xiao-Bing Niu, Shuo Gu, Lu Ji, Bing-Jian Wei, Heng-Bing Wang

**Affiliations:** Department of Urology, The Affiliated Huaian No.1 People's Hospital of Nanjing Medical University, Huaian, Jiangsu, China

## Abstract

Cytotoxic T cell antigen-4 (CTLA-4) is reportedly involved in the development of bladder cancer (BC). This research was designed to address the potential link between the +49A/G polymorphism in CTLA-4 gene and BC susceptibility. In total, 355 BC cases and 435 match controls from Chinese Han individuals were included eventually. The PCR-RFLR method was utilized to screen for this polymorphism. The +49A/G polymorphism was shown to increase the risk of BC. Subgroup analyses showed that this polymorphism was linked to an increased susceptibility to BC among individuals aged < 60 years, smokers and drinkers. Additionally, this polymorphism significantly correlated with tumor node metastasis and tumor size (≥3 cm). To sum up, this study reveals that the CTLA-4 +49A/G polymorphism could increase the risk of BC in Chinese Han people. Further large cohort studies with enough sample sizes are urgently warranted to verify the findings of this present study.

## 1. Introduction

Bladder cancer (BC) is reported to be one of the most common genitourinary tumors [1]. BC is reportedly the ninth most common cancer worldwide, and the incidence rates of BC in men are the highest in Southern and Western Europe, Western Asia, and North America [2]. BC incidence in women is considerably lower than in men, although difference in incidence between the genders varies among countries [2]. Although genetic predisposition, chronic irritation, and certain environmental conditions have been reported as potential risk factors for BC [3–6], the main risk factor is tobacco smoking [7]. GWAS studies identified susceptibility loci for BC [8–10].

Cytotoxic T cell antigen-4 (CTLA-4), homologous to the CD28 protein receptor, negatively regulates the T cell proliferation and activation [11]. By interfering with T cell activation, CTLA-4 hampers antitumor-related immune responses and promotes tumor growth [12]. Zhang et al. observed that disrupting the CTLA-4 expression on CD8^+^ T cells promoted antitumor immune responses in BC [13]. Additionally, CTLA-4 inhibition could suppress the growth of BC [14]. Immunotherapy containing anti-CTLA-4 antibodies showed promise as a curative approach for BC [15].

The CTLA-4 gene is shown to locate on chromosome 2q33. Previous studies have suggested a link between CTLA-4 +49A/G polymorphism and several types of cancer risk, including hepatocellular carcinoma [16], colorectal cancer [17], and osteosarcoma [18]. Two studies investigating the relationship between +49A/G polymorphism and BC susceptibility in different populations yielded inconsistent results [19, 20]. This study was intended to address whether +49A/G polymorphism in CTLA-4 gene was related to BC susceptibility in this population.

## 2. Methods

### 2.1. Participants

This research enrolled 355 BC patients eventually. Details regarding the inclusion criteria for the BC group selection were described in a previous study [21]. During the same period, 435 healthy age- and sex-matched controls receiving a regular health checkup at the same hospital were also recruited. All patients and controls provided written informed consent; clinical information was collected using a structured questionnaire. The study was consented by the Ethics Committee of our hospital and was in line with the Declaration of Helsinki.

### 2.2. Genotyping

By utilization of the QIAamp DNA Blood Mini Kit from QIAGEN, Hilden, Germany, all DNA samples were acquired from EDTA-peripheral blood. The PCR-RFLP method could genotype CTLA-4 +49A/G polymorphism. The PCR primers included 5′-AAGGCTCAGCTGAACCTGGT-3′ and 5′-CTGCTGAAACAAATGAAACCC-3′. To confirm the reproducibility of this method, 10% of the samples were regenotyped, and their consistence was 100%.

### 2.3. Statistical Analysis

The chi-squared (*χ*^2^) test was used to analyze categorical variables; the Student's *t*-test was utilized to determine continuous variables. Hardy-Weinberg equilibrium (HWE) was used to test for CTLA-4 +49A/G polymorphisms among healthy individuals. Logistic regression was to evaluate ORs and their 95% CIs. *P* value of <0.05 indicated statistically significant results. All relevant statistical analyses were performed by the SPSS software package (ver. 22.0; SPSS Inc., Chicago, IL, USA) or the SAS software (version 9.1.3; SAS Institute, Cary, NC, USA).

## 3. Results

### 3.1. Demographic Data

The demographic data of all participants are detailed in Table 1. Information about tumor grade, tumor size, TNM stage, tumor node metastasis, distant metastasis, and histology is also provided in Table 1 for BC patients. We found the incidences of sex (*P* = 0.464) or age (*P* = 0.541) did not differ significantly. However, the smoking and drinking rates were higher in BC patients compared with healthy individuals.

### 3.2. CTLA-4 +49A/G Polymorphism and Risk of BC

The genotype distribution of this polymorphism in healthy individuals was determined by use of the HWE test; no significant enrichment was found. The GG and AG+GG genotypes showed an increased risk for BC (Table 2). These associations still reached significant after adjusting for gender and age. Additionally, the G allele increased the risk of BC (G versus A: OR, 1.39; 95% CI, 1.09-1.77; *P* = 0.008). The subgroup analyses stratified by age, sex, drinking, and smoking are shown in Table 3. Smoking, drinking and age were related to an increased risk of BC, but no significant association was shown in the sex subgroup analysis.

### 3.3. CTLA-4 +49A/G Polymorphism Relates to Certain Clinicopathological Characteristics of BC

We also interpreted the link between this polymorphism and the clinicopathological characteristics of BC patients. We observed that +49A/G polymorphism had a connection with tumor node metastasis and tumor size (Table 4). However, no potential association between CTLA-4 +49A/G polymorphism and tumor grade, distant metastasis, TNM stage, or histology of BC was indicated.

## 4. Discussion

In this study, we found that +49A/G polymorphism elevated the risk of BC in Chinese Han population. Moreover, subgroup analyses suggested that the polymorphism was significantly correlated to age, smoking and drinking. This SNP was shown to have a link with tumor node metastasis and larger tumor size of BC patients.

Recently, Fang et al. designed a meta-analysis exploring the link between CTLA4+49A/G polymorphism and cancer susceptibility. They found that this polymorphism increased the risk of bone, liver, breast, pancreatic, and head and neck cancer; however, they found no association with the risk of gastric, colorectal, renal, or lung cancer [22], suggesting a potential cancer-specific effect. Wang et al. were the first research team to report a link between this SNP and BC susceptibility; their findings revealed that this polymorphism could decrease the risk of BC. After stratifying the BC patients, the associations between the polymorphism and clinical parameters including grade, stage, and histological types were not significant, suggesting that this SNP likely does not contribute to BC development and progression [19]. A subsequent study, however, indicated the presence of +49A/G polymorphism was related to increased susceptibility to BC and that this SNP increased the susceptibility of BC among smokers. However, CTLA-4 +49A/G had no association with tumor stage or grade in BC patients [20]. Our findings suggested that this SNP elevated the risk of BC. The fact that these studies involved BC patients differing in ethnicity (and thus having distinct genetic backgrounds) might explain the discrepant findings. Differences in sample size, clinical heterogeneity of the cohorts, and diversity in the environmental exposure factors and lifestyle among geographical regions might also contribute to the conflicting results.

We obtained a significant relationship between the +49A/G polymorphism and BC susceptibility among smokers and drinkers, suggesting that the presence of this SNP in combination with smoking/drinking may further increase the risk of BC. Furthermore, this SNP was linked with tumor node metastasis and tumor size of BC. We found no relationship between this polymorphism and TNM stage or tumor grade, in line with previous findings [20].

This study had several limitations. First, the findings of this study could not be extrapolated to other races with different genetic backgrounds. Second, the cohort size was small, especially after stratification of the patients. Third, we focused on a SNP in the CTLA-4 gene, thus ignoring the impact of potential interactions among polymorphisms. Fourth, the impact of +49A/G polymorphism on the transcription and translation of CTLA-4 gene was not investigated. Fifth, data on certain environmental factors that BC patients are exposed to were not available for further analysis.

To sum up, this study observes that CTLA-4 +49A/G polymorphism increases the risk of BC in Chinese individuals. The findings of this study require validation in future large cohort studies.

## Figures and Tables

**Table 1:** Patient demographics and risk factors in bladder cancer.

Variable	Cases (*n* = 355)	Controls (*n* = 435)	*P*
Age (years)	60.81 ± 10.57	61.25 ± 9.73	0.541
Sex			0.464
Male	303 (85.4%)	363 (83.4%)	
Female	52 (14.6%)	72 (16.6%)	
Smoking			<0.001
Yes	251 (70.7%)	180 (41.4%)	
No	104 (29.3%)	255 (58.6%)	
Drinking			<0.001
Yes	233 (65.6%)	173 (39.8%)	
No	122 (34.4%)	262 (60.2%)	
Tumor grade			
High (G2 + G3)	227 (63.9%)		
Low (G1)	128 (36.1%)		
Tumor size (cm)			
<3	263 (74.1%)		
≥3	92 (25.9%)		
TNM stage			—
I	78 (22.0%)	—	—
II	100 (28.2%)		
III	104 (29.3%)		
IV	73 (20.6%)		
Tumor node metastasis			
Yes	108 (30.4%)		
No	247 (69.6%)		
Distant metastasis		—	—
M0	332 (93.5%)	—	—
M1	23 (6.5%)	—	—
Histology			
Papillary	291 (82.0%)		
Nonpapillary	64 (18.0%)		

**Table 2:** Logistic regression analysis of associations between CTLA-4 +49A/G polymorphism and risk of bladder cancer.

Genotype	^#^Cases (*n* = 355)		^#^Controls (*n* = 435)		OR (95% CI)	*P*	^∗^OR (95% CI)	^∗^ *P*
	*n*	%	*n*	%				
AA	206	58.2%	288	66.3%	1.00		1.00	
AG	127	35.9%	132	30.5%	1.35 (0.99-1.82)	0.055	1.35 (1.00-1.82)	0.054
GG	21	5.9%	14	3.2%	**2.10 (1.04-4.22)**	**0.038**	**2.10 (1.04-4.23)**	**0.038**
AG + GG	148	41.8%	146	33.7%	**1.42 (1.06-1.89)**	**0.019**	**1.42 (1.06-1.90)**	**0.018**
AA + AG	333	94.1%	420	96.8%	1.00		1.00	
GG	21	5.9%	14	3.2%	1.89 (0.95-3.78)	0.071	1.90 (0.95-3.79)	0.070
A allele	539	76.13%	708	81.6%	1.00		1.00	
G allele	169	23.87%	160	18.4%	**1.39 (1.09-1.77)**	**0.008**	—	—

^#^The genotyping was successful in 354 cases and 434 controls for CTLA4 +49A/G; bold values are statistically significant (*P* < 0.05). ^∗^Adjustments for age and sex.

**Table 3:** Stratified analyses between CTLA-4 +49A/G polymorphism and the risk of bladder cancer.

Variable	Case/control			AG vs. AA OR (95% CI); *P*	GG vs. AA OR (95% CI); *P*	GG vs. AG + AA OR (95% CI); *P*	GG + AG vs. AA OR (95% CI); *P*
	AA	AG	GG				
Sex							
Male	178/239	107/111	17/12	1.29 (0.93-1.80); 0.124	1.90 (0.89-4.08); 0.099	1.74 (0.82-3.70); 0.151	1.35 (0.99-1.86); 0.060
Female	28/49	20/21	4/2	1.67 (0.77-3.60); 0.193	3.50 (0.60-20.34); 0.301	2.92 (0.51-16.56); 0.404	1.83 (0.87-3.82); 0.109
Smoking							
Yes	136/122	99/49	15/8	**1.81 (1.19-2.76); 0.006**	**1.68 (0.69-4.11); 0.253**	1.36 (0.57-3.29); 0.489	**1.79 (1.20-2.68); 0.004**
No	70/166	28/83	6/6	0.80 (0.48-1.33); 0.392	2.37 (0.74-7.60); 0.147	2.54 (0.80-8.07); 0.102	0.91 (0.56-1.47); 0.689
Drinking							
Yes	130/110	84/58	18/5	1.23 (0.81-1.87); 0.343	**3.05 (1.10-8.47); 0.033**	**2.83 (1.03-7.77); 0.044**	1.37 (0.92-2.05); 0.127
No	76/178	43/74	3/9	1.36 (0.86-2.16); 0.191	0.78 (0.21-2.96); 0.967	0.71 (0.19-2.66); 0.839	1.30 (0.83-2.03); 0.256
Age (years)							
<60	87/126	54/53	13/9	1.48 (0.93-2.35); 0.103	2.09 (0.86-5.11); 0.105	1.83 (0.76-4.41); 0.176	**1.57 (1.01-2.43); 0.046**
≥60	119/162	73/79	8/5	1.26 (0.85-1.87); 0.257	2.18 (0.70-6.83); 0.182	2.01 (0.65-6.24); 0.228	1.31 (0.89-1.93); 0.167

Bold values are statistically significant (*P* < 0.05).

**Table 4:** The associations between CTLA-4 +49A/G polymorphism and clinical characteristics of bladder cancer.

Characteristics	Genotype distributions			
	AA	AG	GG	AG + GG
Tumor grade				
High/low	139/67	77/50	11/10	88/60
OR (95% CI); *P* value	1.00 (reference)	0.74 (0.47-1.18); 0.204	0.53 (0.22-1.31); 0.164	0.71 (0.46-1.10); 0.121
Tumor size				
≥3/<3	42/164	39/88	11/10	50/98
OR (95% CI); *P* value	1.00 (reference)	**1.73 (1.04-2.87); 0.033**	**4.30 (1.71-10.79); 0.001**	**1.99 (1.23-3.22); 0.005**
TNM stage				
III + IV/I + II	96/110	70/57	11/10	81/67
OR (95% CI); *P* value	1.00 (reference)	1.41 (0.90-2.19); 0.131	1.26 (0.51-3.10); 0.613	1.39 (0.90-2.12); 0.131
Tumor node metastasis				
Yes/no	55/151	41/86	12/9	53/95
OR (95% CI); *P* value	1.00 (reference)	1.31 (0.81-2.12); 0.274	**3.66 (1.46-9.17); 0.004**	1.53 (0.97-2.42); 0.066
Distant metastasis				
M1/M0	12/194	8/119	3/18	11/137
OR (95% CI); *P* value	1.00 (reference)	1.09 (0.43-2.74); 0.860	2.69 (0.70-10.44); 0.137	1.30 (0.56-3.03); 0.545
Histology				
Papillary/nonpapillary	166/40	106/21	19/2	125/23
OR (95% CI); *P* value	1.00 (reference)	1.22 (0.68-2.18); 0.509	2.29 (0.51-10.23); 0.414	1.31 (0.75-2.30); 0.347

Bold values are statistically significant (*P* < 0.05).

## Data Availability

The data of this study can be obtained from the corresponding author upon request.
